# Natural genetic variation in the pheromone production of *C. elegans*

**DOI:** 10.1073/pnas.2221150120

**Published:** 2023-06-20

**Authors:** Daehan Lee, Bennett W. Fox, Diana Fajardo Palomino, Oishika Panda, Francisco J. Tenjo, Emily J. Koury, Kathryn S. Evans, Lewis Stevens, Pedro R. Rodrigues, Aiden R. Kolodziej, Frank C. Schroeder, Erik C. Andersen

**Affiliations:** ^a^Department of Molecular Biosciences, Northwestern University, Evanston, IL 60208; ^b^Department of Biology, Kyung Hee University, Seoul 02447, Republic of Korea; ^c^Department of Biological Sciences, Sungkyunkwan University, Suwon 16419, Republic of Korea; ^d^Boyce Thompson Institute, Cornell University, Ithaca, NY 14850; ^e^Department of Chemistry and Chemical Biology, Cornell University, Ithaca, NY 14850; ^f^Tree of Life, Wellcome Sanger Institute, Cambridge CB10 1SA, United Kingdom

**Keywords:** *C. elegans*, pheromone, natural variation, ascaroside, fatty acid metabolism

## Abstract

From bacterial quorum sensing to human language, communication is essential for social interactions. Nematodes produce and sense pheromones to communicate among individuals and respond to environmental changes. These signals are encoded by different types and mixtures of ascarosides, whose modular structures further enhance the diversity of this nematode pheromone language. Interspecific and intraspecific differences in this ascaroside pheromone language have been described previously, but the genetic basis and molecular mechanisms underlying the variation remain largely unknown. Here, we analyzed natural variation in the production of 44 ascarosides across 95 wild *Caenorhabditis elegans* strains using high-performance liquid chromatography coupled to high-resolution mass spectrometry. We discovered wild strains defective in the production of specific subsets of ascarosides (*e.g.*, the aggregation pheromone icas#9) or short- and medium-chain ascarosides, as well as inversely correlated patterns between the production of two major classes of ascarosides. We investigated genetic variants that are significantly associated with the natural differences in the composition of the pheromone bouquet, including rare genetic variants in key enzymes participating in ascaroside biosynthesis, such as the peroxisomal 3-ketoacyl-CoA thiolase, *daf-22*, and the carboxylesterase *cest-3*. Genome-wide association mappings revealed genomic loci harboring common variants that affect ascaroside profiles. Our study yields a valuable dataset for investigating the genetic mechanisms underlying the evolution of chemical communication.

## Significance

Pheromones are signaling molecules used for chemical communication in social interactions. Although diverse pheromone classes from diverse species have been identified, little is known about how chemical languages evolve. To study the genetic basis underlying natural diversity in animal chemical language, we analyzed the secreted pheromones of 95 whole-genome sequenced wild *Caenorhabditis elegans* strains. We characterized the genetic architectures underlying natural differences in the production of 44 ascarosides, which represent the nematode’s primary pheromone class. Our study uncovered “hot spot loci” that broadly impact ascaroside production, as well as inverse correlations between two major classes of ascarosides. Our findings provide insights into how metabolism and chemical communication are coupled in evolution.

**Fig. 1.**
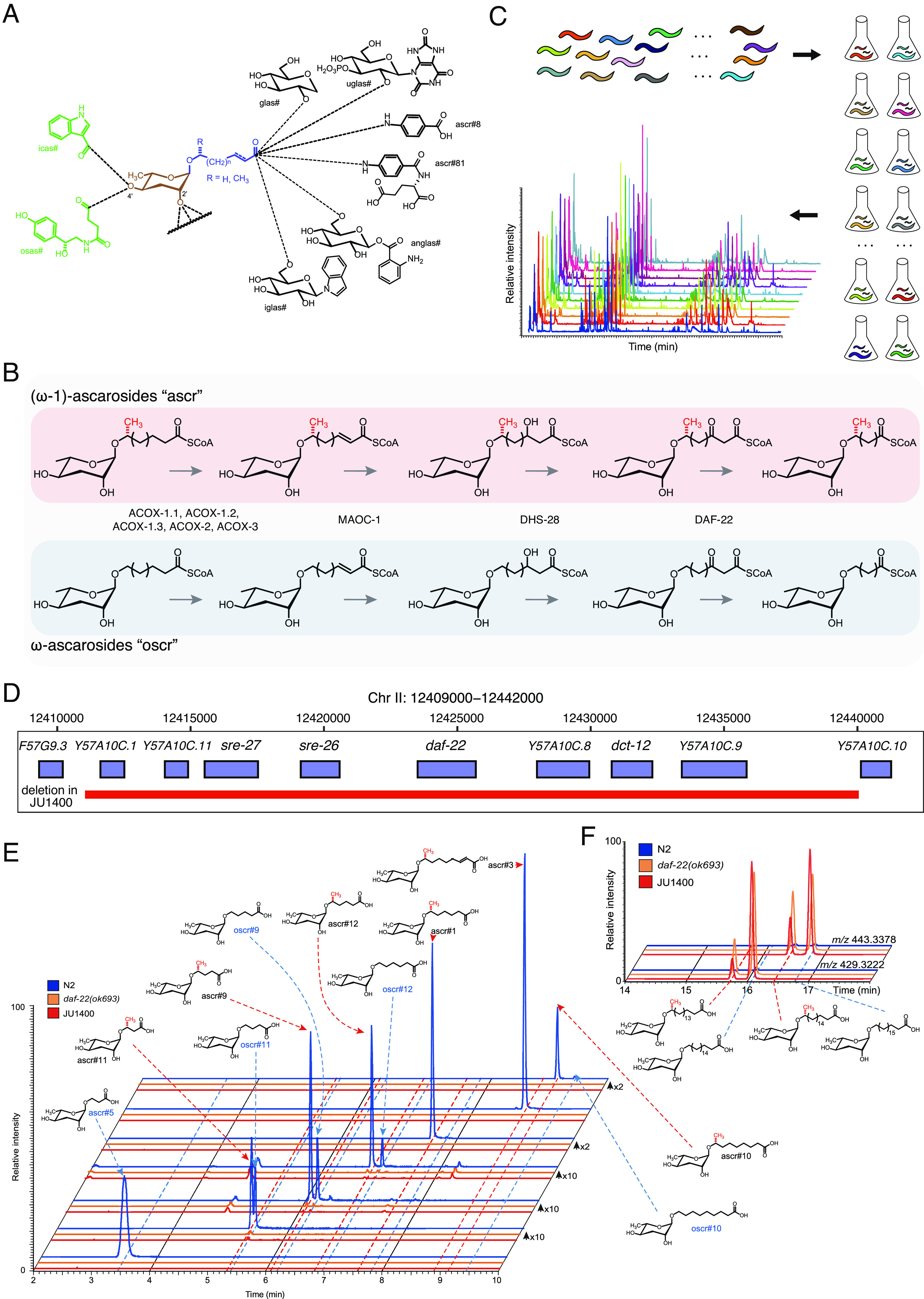
A natural deletion in the *daf-22* gene was found in a pheromone-deficient wild *Caenorhabditis elegans* strain. (*A*) Chemical structures of the ascarosides. Simple ascarosides consist of the dideoxy sugar ascarylose (brown) and an FA–like side chain of varying lengths (blue). Modular ascarosides incorporate additional building blocks from other primary metabolic pathways, *e.g*., indole 3-carboxylic acid (icas#, green) derived from tryptophan, the neurotransmitter octopamine (osas#, green), and a variety of C-terminal modified ascarosides, including folate derivatives (ascr#8, ascr#81) and several glucosides (black) (12). (*B*) Chain shortening of ascarosides by enzymes in the peroxisomal β-oxidation pathway. Ascarosides of the two classes (“ascr” and “oscr”) are derived from very long-chain precursors. (*C*) Schematic of the ascaroside profiling experiments, in which 95 *C. elegans* strains were grown in liquid cultures, and the conditioned media extracted and analyzed by HPLC-HRMS, then correlated with genomic data to identify QTL underlying natural differences in pheromone production. (*D*) A 29,011 bp natural deletion in the JU1400 strain encompassing *daf-22* and seven additional genes. (*E*) Extracted ion chromatograms (EICs) corresponding to several short- and medium-chain ascarosides, as indicated, in the N2 strain (wild-type, WT), *daf-22(ok693)*, and the JU1400 strain *exo*-metabolome extracts from synchronized adults. *Y* axis are scaled as indicated to clearly show lower-intensity metabolites. (*F*) EICs for *m/z* 429.3222 and 443.3378, corresponding to precursor ascarosides with C_18_ and C_19_ sidechains, respectively, in the N2 strain (WT), *daf-22(ok693)*, and the JU1400 strain *exo*-metabolome extracts from synchronized adults.

“Pheromone” is an informative chemical or mixture of chemicals that an organism produces and secretes into the environment, affecting the behavior, physiology, and development of other individuals. Nematodes use pheromones called ascarosides (1, 2), which consist of the dideoxy sugar, ascarylose, linked to diverse fatty acid–like (FA–like) side chains, and can be decorated with diverse derivatives of amino acids, folate, and other primary metabolites (3) (Fig. 1*A*). The nematode sensory system perceives distinct combinations and concentrations of ascarosides (4), which in turn modulate a variety of biological processes, including developmental plasticity, social and sexual behaviors, olfactory learning, stress response, reproduction, and longevity (1, 5–11).

Studies of the model nematode *Caenorhabditis elegans* have uncovered a complex network of ascaroside biosynthetic pathways. One of the key biochemical reactions in ascaroside production is the iterative shortening of the FA–like side chains by peroxisomal β-oxidation (Fig. 1*B*). Mutations in peroxisomal β-oxidation genes impair the production of functional short- and medium-chained ascarosides that control development and behavior (13–17). Analysis of peroxisomal β-oxidation mutants, in particular *daf-22(ok693)*, revealed an accumulation of long-chained precursors with both odd and even numbers of carbons in the side chain (16). Although the roles of many genes involved in the production of ascarosides have been characterized (*e.g*., *daf-22*, *dhs-28*, *maoc-1*, and acyl-CoA oxidase orthologs) (16, 18–21), the upstream pathway that produces long-chained precursor ascarosides is largely unknown.

Ascaroside pheromones are a universal nematode chemical language found across diverse parasitic and free-living species (2), but the repertoire of ascaroside pheromones varies from species to species. For example, a dimeric ascaroside discovered in *Pristionchus pacificus*, dasc#1, regulates the mouth-form dimorphism underlying its facultative predatory lifestyle (22). In addition, intraspecific quantitative variation in pheromone production has been observed in both *C. elegans* and *P. pacificus* species (2, 23, 24). These discoveries suggest that ascaroside biosynthetic pathways vary within and across species. In line with the natural variation in pheromone production, natural differences in pheromone responses have been demonstrated as well (23, 25–28). Here, we characterized the genetic basis of natural variation in *C. elegans* pheromone production. We analyzed the secreted metabolites from 95 wild *C. elegans* strains and profiled their pheromone bouquets by measuring relative abundances of 44 different ascarosides. Our quantitative genetic analysis of heritable variation in ascaroside production revealed diverse links between natural differences in metabolism and chemical communication of the species.

## Results

### A Peroxisomal β-Oxidation Gene Is Deleted in a Pheromone-Less Wild *C. elegans* Strain.

To investigate the intraspecific variation in *C. elegans* pheromone production, we analyzed the *exo*-metabolomes of 95 wild strains using high-performance liquid chromatography coupled to high-resolution mass spectrometry (HPLC-HRMS) (Fig. 1*C* and *Methods*). Because ascaroside biosynthesis is affected by diverse factors including sex, developmental timing, and nutrition (29), we chose to analyze the *exo*-metabolomes of synchronized hermaphrodites at the young adult stage. Among thousands of detected metabolites, we identified and quantified 44 ascarosides (*Methods* and *SI Appendix*, Fig. S1). Unexpectedly, we found that short- and medium-chain ascarosides were almost completely absent in a single wild strain (JU1400), suggesting that key steps in ascaroside biosynthesis could be impaired in this strain. Previously, mutations in peroxisomal β-oxidation genes (*e.g*., *daf-22*, *dhs-28, maoc-1*) (Fig. 1*B*) were shown to abolish the production of short- and medium-chain ascarosides (16). To investigate whether the JU1400 strain has an impaired peroxisomal β-oxidation pathway, we performed a de novo assembly of this genomic region and identified a large deletion (29 kb) in the *daf-22* locus, which completely removes *daf-22* and seven neighboring genes (Fig. 1*D*). Consistent with this deletion, the metabolic phenotype of the JU1400 strain closely resembled that of *daf-22(ok693)* loss-of-function mutants, which lack short- and medium-chain ascarosides and instead accumulate large amounts of long-chain precursor ascarosides (Fig. 1 *E* and *F*). Intriguingly, the ratio of very long-chain (ω-1)- to ω-ascarosides in *daf-22(ok693)* and JU1400 was similar (Fig. 1*F*), suggesting that the ratio of these precursors is controlled independently of peroxisomal β-oxidation. We extended our analysis of the *daf-22* locus to 538 wild genomes (30) but did not find the same deletion nor any nonsense mutation in any other wild strains, suggesting that the loss of a β-oxidation gene that leads to the severe impairment of ascaroside production is rare in the natural *C. elegans* population.

### The Pheromone Bouquet Varies among Wild *C. elegans* Strains.

**Fig. 2.**
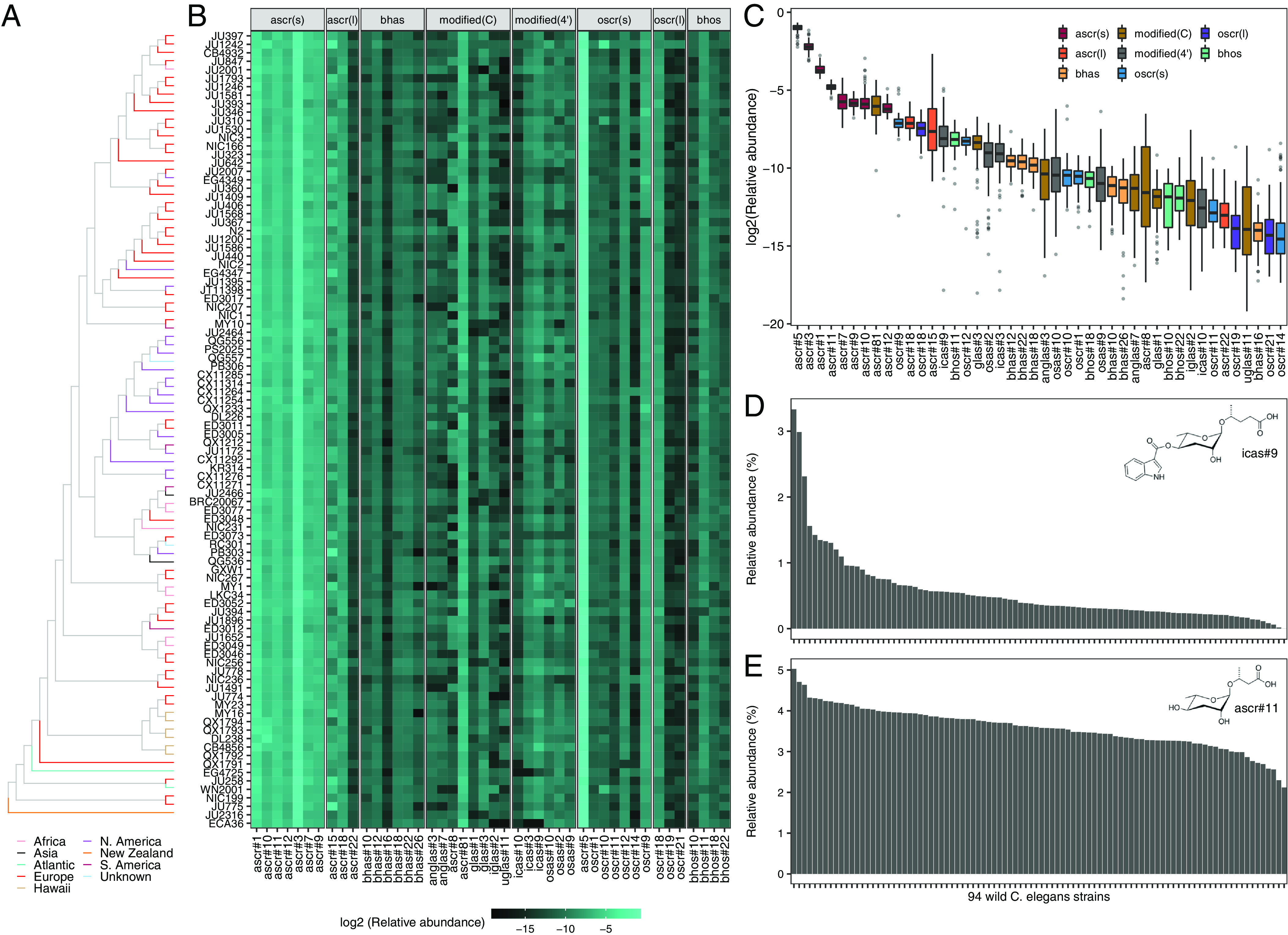
Pheromone bouquet varies across wild *C. elegans* strains. (*A*) Neighbor-joining tree of 94 wild *C. elegans* strains analyzed in this study (except the JU1400 strain) generated from 963,027 biallelic segregating sites. The terminal branch is colored by the strain’s geographic origin. (*B*) A heatmap showing relative abundances of 44 ascarosides. Each row represents one of the 94 wild strains, ordered by genome-wide relatedness. Ascarosides are grouped by structural similarity. (*C*) Tukey box plots of the relative abundances of 44 ascarosides across 94 wild *C. elegans* strains are shown with outlier strain data points plotted. The horizontal line in the middle of the box is the median, and the box denotes the 25th to 75th quantiles of the data. The vertical line represents the 1.5× interquartile range. Box plots are colored by structural properties that were used for ascaroside grouping in *B*. (*D* and *E*) Bar plots showing relative abundances of icas#9 (*D*) and ascr#11 (*E*) across 94 wild *C. elegans* strains, ordered by relative abundance.

*C. elegans* produces and releases a diverse collection of ascarosides with different lengths of FA side chains as well as other types of modifications (Fig. 1*A* and *SI Appendix*, Table S1). Based on the HPLC-HRMS data, we compared the composition and abundances of pheromones among 94 wild *C. elegans* strains (Fig. 2 *A* and *B* and Dataset S1), excluding the JU1400 strain that lacks the majority of short- and medium-chain ascarosides. For each strain, we calculated the intensity of each ascaroside relative to the sum of the 44 measured ascarosides (henceforth, referred to as relative abundance). On average, we found that two major ascarosides, ascr#5 and ascr#3, which are derived from the ω and ω-1 pathways, comprised 51.2% and 21.4% of measured ascarosides, respectively (Fig. 2*C*). The rest of the identified ascarosides comprised 0.004 to 7.4%, and the relative abundances of each ascaroside varied from strain to strain, though to different extents (Fig. 2 *B* and *C* and *SI Appendix*, Fig. S2). For example, a pheromone that promotes aggregation at picomolar concentrations, referred to as icas#9 (indole-3-carboxylic acid ascarosides) (8, 31), was not detected in the ECA36 strain but it comprised 3.3% of total ascarosides in the CB4856 strain (Fig. 2*D*). Notably, ECA36 possesses a nonsense mutation in *cest-3*, which encodes the enzyme required for 4′ attachment of indole 3-carboxylic acid to the ascarylose core (32). By contrast, ascr#11 was much less variable than icas#9 across the 94 wild strains, as its relative abundance ranged from 2.1 to 5% (Fig. 2*E*).

**Fig. 3.**
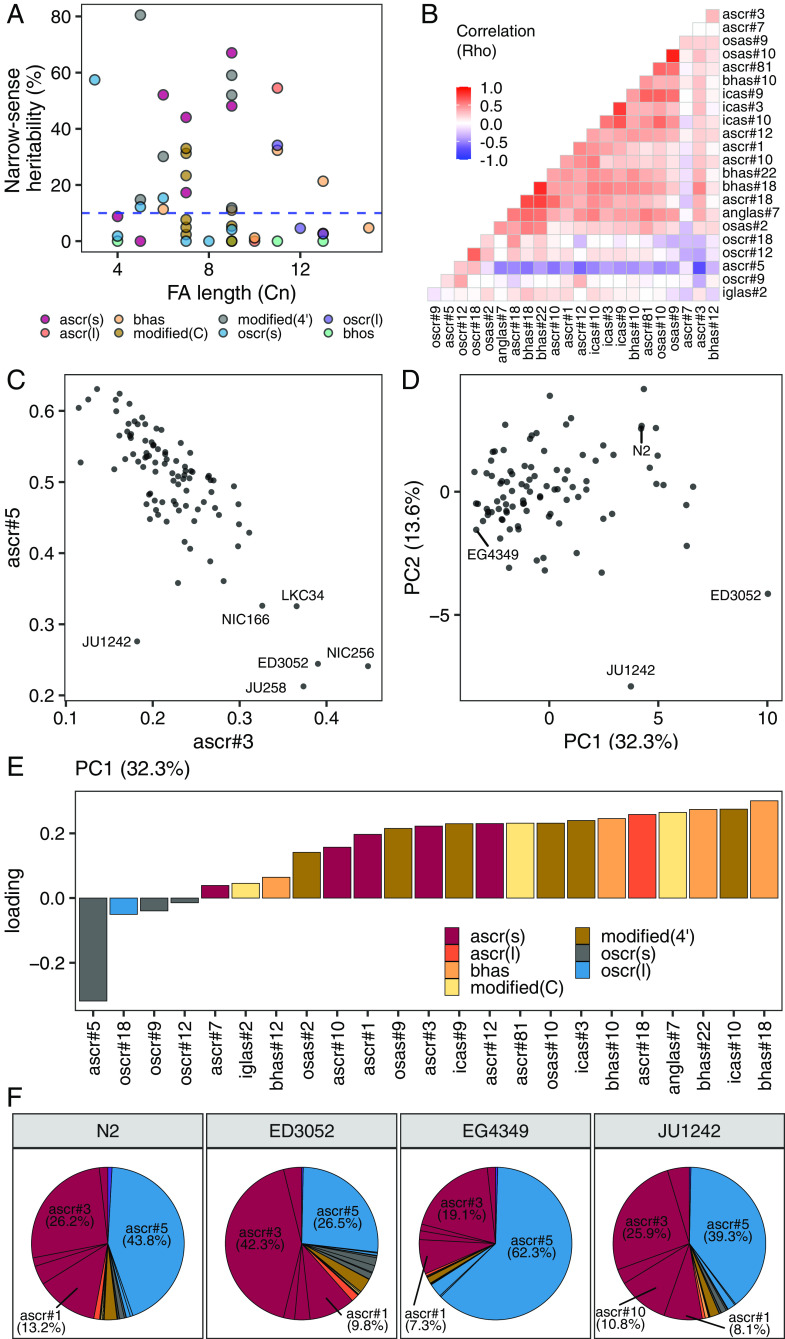
Relative abundances of ω- and (ω-1)-ascarosides are inversely correlated. (*A*) Narrow-sense heritabilities of 44 different ascarosides are shown. Each point corresponds to one of the 44 ascarosides and is colored by the structural property. Cn: Number of carbons. The blue dashed horizontal line shows the heritability threshold (10%) for downstream analyses. The length of the FA side chain is shown on the *x* axis. (*B*) A heatmap showing correlation (Spearman’s rho) of relative abundance traits between two different ascarosides. (*C*) A scatter plot showing relative abundances of ascr#3 (*x* axis) and ascr#5 (*y* axis). Each point corresponds to one of the 94 wild *C. elegans* strains. (*D*) Plots of the 94 wild strains showing their values for each of the two significant axes of variation, as determined by PCA of the ascaroside pheromone bouquet. (*E*) Relative abundance trait loadings of the first PC that explains up to 32.3% of the total variance in the trait data are shown. (*F*) Pie charts showing the relative abundances of 23 ascarosides with high heritability (>10%) for four wild *C. elegans* strains labeled in *D* are shown. (*E* and *F*) Bar plots and pie charts are colored by the structural properties as in *A*.

To investigate the genetic contributions to natural variation in ascaroside abundances, we analyzed the heritabilities for relative abundance traits of 44 ascarosides. We found that the narrow-sense heritabilities (*h^2^*) of 44 ascaroside abundance traits ranged from 0 to 80% (Fig. 3*A*). Variation in the icas#9 abundance trait exhibited the highest heritability (80%), followed by ascr#10 (67%), icas#10 (59%), and ascr#5 (57%). All icas# showed high heritabilities (>50%), whereas differences in β-hydroxylated ω-ascarosides (bhos#10, bhos#11, bhos#18, bhos#22) were not explained by additive genetic factors (*h^2^* = 0%). To focus on genetic differences in pheromone production, we chose 23 ascarosides that showed at least 10% of total additive genetic variance for further study.

We analyzed the correlation patterns among these 23 heritable traits and found that negative correlations are prevalent between ω-ascarosides and (ω-1)-ascarosides. For example, the most abundant ω-ascaroside (ascr#5) showed negative correlations with all (ω-1)-ascarosides but positive correlations with all ω-ascarosides (oscr#9, oscr#12, oscr#18) (Fig. 3*B*). The strongest correlations (Spearman’s *rho* = -0.81) were observed between ascr#5 and the most abundant (ω-1)-ascaroside (ascr#3) (Fig. 3 *B* and *C*). Furthermore, the ratio between ascr#3 and ascr#5 was remarkably heritable (*h^2^*= 82.1%), which was even higher than the *h^2^* values of the relative abundance traits for ascr#3 (48.1%) or ascr#5 (57.4%). We observed a similar trend using principal component analysis (PCA) of the 23 heritable traits (Fig. 3 *D*–*F* and *Methods*). The first principal component (PC) that explained 32.3% of the variance in the dataset had negative loadings for all ω-ascarosides (ascr#5, oscr#9, oscr#12, oscr#18) and positive loadings for all (ω-1)-ascarosides (Fig. 3*E*). Because ω- and (ω-1)-ascarosides are derived from parallel β-oxidation of very-long chain precursors (29), these results suggest that the relative amounts of ω- and (ω-1)-starting materials vary across the species.

To examine the biological significance of the (ω-1)- to ω-ascaroside ratio, we measured the correlation between this trait and an ascr#5-induced dauer formation trait (27). We found that the ascr#3:ascr#5 ratio is weakly correlated (Spearman’s *rho* = 0.192) with ascr#5-induced dauer formation across 61 wild strains. Notably, this weak correlation is largely driven by four outlier strains (JU258, ED3052, LKC34, and NIC166) that exhibit a high ascr#3-to-ascr#5 ratio (*SI Appendix*, Fig. S3).

### A Common Genomic Locus Underlies Variation in Ascaroside Biosynthesis.

**Fig. 4.**
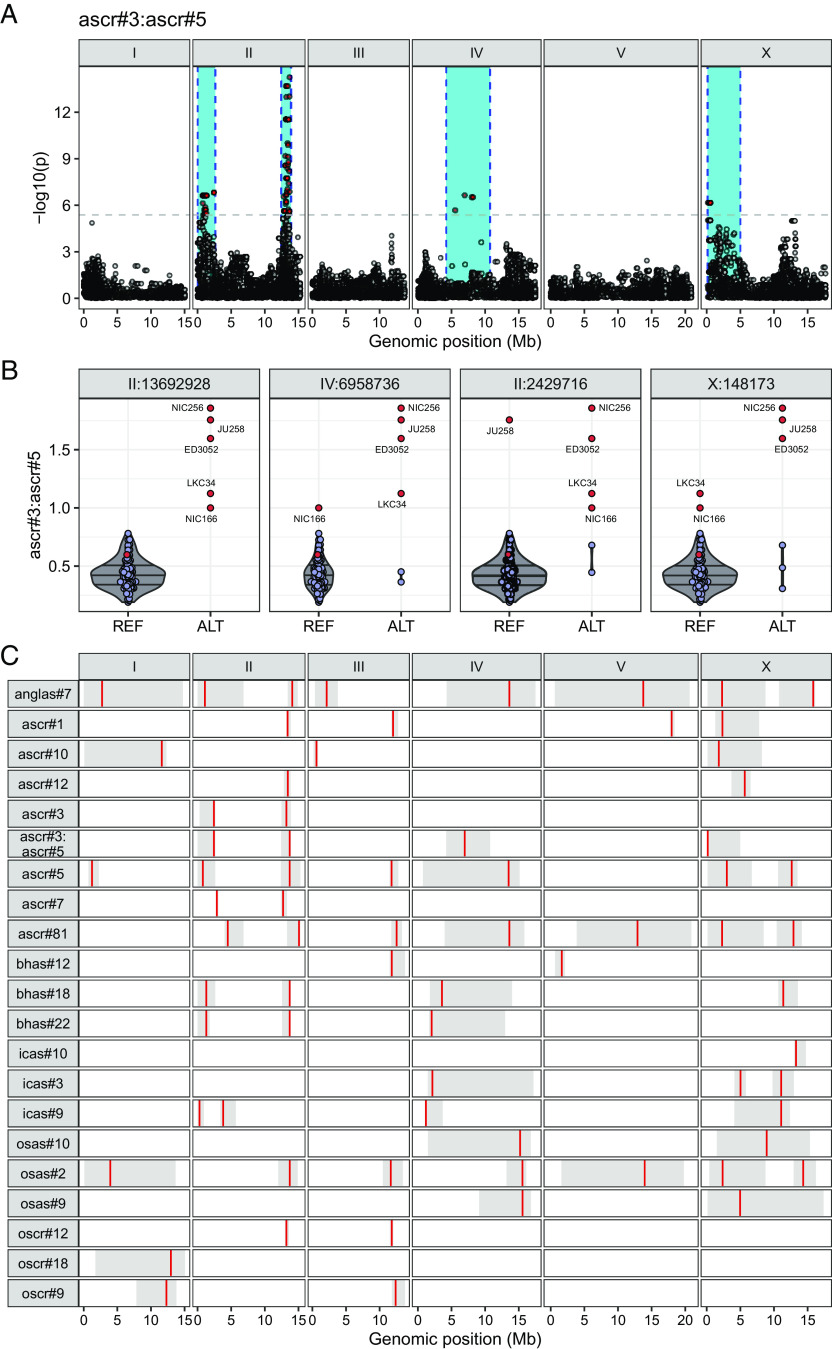
QTL hot spots underlie natural variation in various ascarosides. (*A*) Manhattan plot for GWA mapping of the ascr#3:ascr#5 ratio trait. Each dot represents a single-nucleotide variant (SNV) that is present in at least 5% of the 94 wild strains. The genomic position in Mb, separated by chromosome, is plotted on the *x* axis, and the statistical significance of the correlation between genotype and phenotype is plotted on the *y* axis. The dashed horizontal line denotes the Bonferroni-corrected *P*-value threshold using independent markers correcting for LD (genome-wide eigen-decomposition significance threshold). SNVs are colored red if they pass this threshold. The region of interest for each QTL is represented by a cyan rectangle. (*B*) Beeswarm plots of phenotypes split by peak marker position of the four QTL from *A*. Each dot corresponds to the phenotype of an individual strain, which is plotted on the *y* axis. Strains are grouped by their genotype at each peak QTL position. Dots for the reference N2 strain and five high trait-value strains are colored red. (*C*) A summary plot showing the GWA mapping results including location and range of QTL for relative abundance traits for 20 ascarosides with high trait heritability (>10%) and the ascr#3:ascr#5 ratio trait. Each red bar corresponds to the peak position of the QTL and each gray box represents the region of interest for each QTL.

To characterize genomic loci underlying observed natural differences in the composition of the pheromone bouquet, we performed genome-wide association (GWA) mappings (*SI Appendix*, Table S2). We identified four quantitative trait loci (QTL) from the mapping of ascr#3:ascr#5 ratio trait (Fig. 4 *A* and *B*), including the most significant genomic region on the right arm of chromosome II (peak marker at II:13,692,928), which explained 71.8% of the phenotypic variance. Specifically, five wild strains (ED3052, JU258, LKC34, NIC166, and NIC256) had high ascr#3:ascr#5 ratios (≥1) and the nonreference (ALT) allele at the peak position (Fig. 4*B*). We also performed GWA mapping for heritable (>10%) relative abundance traits of 23 ascarosides and identified QTL from 20 traits (Fig. 4*C*). The ascr#3:ascr#5 trait QTL overlapped with QTL that were mapped for relative abundance traits; ChrIIR-QTL overlapped with 11 ascarosides (anglas#7, ascr#1, ascr#3, ascr#5, ascr#7, ascr#12, ascr#81, bhas#18, bhas#22, osas#2, and oscr#12) and three other QTL (ChrIIL-QTL, ChrIV-QTL, and ChrX-QTL) also overlapped with QTL from other relative abundance traits (Fig. 4*C*). These results together suggest that shared genomic loci (“QTL hot spots”) harbor variants with a broad impact on ascaroside biosynthesis.

**Fig. 5.**
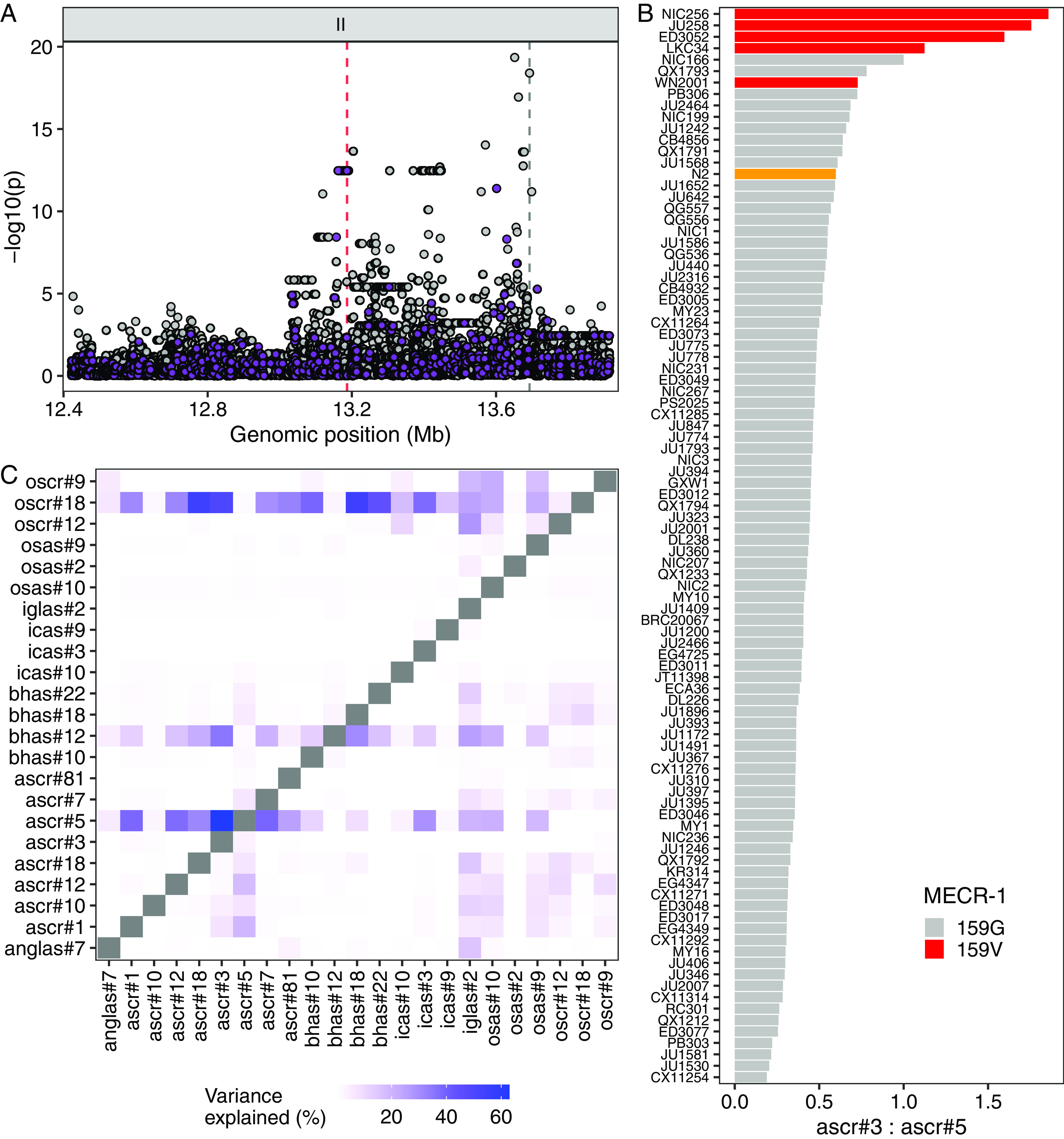
A *mecr-1* variant is associated with natural differences in ascaroside production. (*A*) Fine mapping of the ChrIIR-QTL (II:12,422,412-13,692,928) for the ascr#3:ascr#5 ratio trait is shown. Each dot represents an SNV that is present in at least 5% of the 94 wild strains. The association between the SNV and ascr#3:ascr#5 trait value is shown on the *y* axis, and the genomic position of the SNV is shown on the *x* axis. SNVs with high or moderate impact inferred from SnpEff are colored purple. (*B*) A bar plot for the ascr#3:ascr#5 trait value of 94 wild *C. elegans* strains. The reference N2 strain is colored orange, and wild strains with the MECR-1(G159V) variant are colored red. (*C*) A heatmap showing amounts of variance explained by the MECR-1(G159V) variant for pairwise ratio traits of 23 ascarosides with high trait heritability.

We investigated the QTL hot spot spanning 1.49 Mb on the right arm of chromosome II (ChrIIR-QTL), which explained the largest fraction of variance for the ascr#3:ascr#5 ratio trait and also was mapped for the largest number of relative abundance traits. To identify a quantitative trait gene underlying this hot spot, we performed a fine-mapping for the ascr#3:ascr#5 ratio trait (Fig. 5*A*). Among 9,907 genetic variants in 290 genes across the QTL (Dataset S2), we prioritized genetic variants that were predicted to disrupt protein function (i.e., missense, frameshift). Four single-nucleotide variants (SNVs) were found to be equally and significantly associated with the phenotypic variation (−log_10_*p* = 12.469). Among four genes (*moe-3, W09H1.1*, *W09H1.3*, and *mecr-1*) impacted by these SNVs, we focused on *mecr-1* because of its predicted involvement in fatty acid metabolism, which is upstream of ascaroside biosynthesis and therefore potentially related to the ascr#3:ascr#5 ratio trait. The gene *mecr-1* encodes a mitochondrial trans-2-enoyl-CoA reductase, a key enzyme in mitochondrial FA synthesis (mtFAS) (33), whose potential interactions with ascaroside biosynthesis have not been described previously. We found that four of the five wild strains with high ascr#3:ascr#5 ratios (ED3052, JU258, LKC34, and NIC256) harbored the G159V missense variant in *mecr-1* (Fig. 5*B*), suggesting that this allele could cause increased production of ascr#3, a reduction of ascr#5, or both.

We extended our analysis to the association between the *mecr-1* variant and pairwise ratio traits of all the 23 analyzed ascarosides. We found that the MECR-1(G159V) variant explained much of the phenotypic variance for many traits (Fig. 5*C*). Specifically, the MECR-1(G159V) variant was frequently associated with differences between ω-ascarosides (ascr#5, oscr#9, oscr#12, oscr#18) and (ω-1)-ascarosides (e.g., ascr#1, ascr#3, ascr#7, bhas#18, icas#3, osas#2), suggesting that this variant could affect the balance between the two parallel ascaroside production pathways. In addition, the relative ratio traits between bhas#12, an (ω-1)-oxygenated β-hydroxy ascaroside, and many (ω-1)-ascarosides were associated with the MECR-1(G159V) variant. Relative abundances of iglas#2, osas#9, and osas#10 to many other ascarosides [both ω-ascarosides and (ω-1)-ascarosides] were also associated with this same variant. Taken together, these results suggested that the *mecr-1* variant might broadly affect ascaroside biosynthesis pathways.

### Natural Genetic Variants in the mtFAS Pathway Are Associated with the Variation in Ascaroside Biosynthesis.

To test whether the MECR-1(G159V) variant underlies observed differences in ascaroside production, we performed allele-replacement experiments. Using CRISPR-Cas9 to edit the *mecr-1* locus, we tried to substitute the glycine at position 159 with a valine in the N2 strain and the valine at position 159 with a glycine in the ED3052 strain (*Methods*). We successfully generated N2 MECR-1(G159V), but we failed to replace the valine with glycine in the ED3052 strain after extensive trials (no. of injected animals = 63). We also failed to introduce the same valine-to-glycine edit in the NIC256 strain (no. of injected animals = 43). Although our results suggest a potential genetic incompatibility of glycine at this residue in these genetic backgrounds, possibly from uncharacterized genetic interactions with other alleles, we cannot conclusively rule out other technical reasons [e.g., inefficient homology-directed repair (HDR)] for our inability to create the desired edit. We grew allele-replacement strains in the N2 background and examined the phenotypic effects of the MECR-1(G159V) variant on the profile of excreted ascarosides. We found that two independent N2 MECR-1(G159V) strains, ECA2818 and ECA2834, produced an N2-like pheromone blend, as measured by the ascr#3:ascr#5 ratio, whereas the ED3052 and NIC256 strains grown in parallel reproduced the altered ascr#3:ascr#5 ratio trait (*SI Appendix*, Fig. S4).

Because the MECR-1(G159V) allele–replacement strains did not recapitulate the QTL effect, we hypothesized that multiple linked alleles together might contribute to the QTL effect. Notably, ALT alleles at the peak markers of all four ascr#3:ascr#5 ratio QTL (II:2,429,716; II:13,692,928; IV:6,958,736, and X:148,173) were associated with greater trait values (Fig. 4*B*), and these alleles displayed strong linkage disequilibrium (LD) (*SI Appendix*, Fig. S5). Specifically, two QTL that are on opposite arms of chromosome II (ChrIIL-QTL-ChrIIR-QTL) showed high LD (*r^2^* = 0.509). Surprisingly, we detected the same level of LD between QTL on different chromosomes (ChrIIR-QTL-ChrV-QTL, *SI Appendix*, Fig. S5). These intrachromosomal and interchromosomal LD might reflect genetic interactions between MECR-1(G159V) and other uncharacterized alleles in pheromone production.

**Fig. 6.**
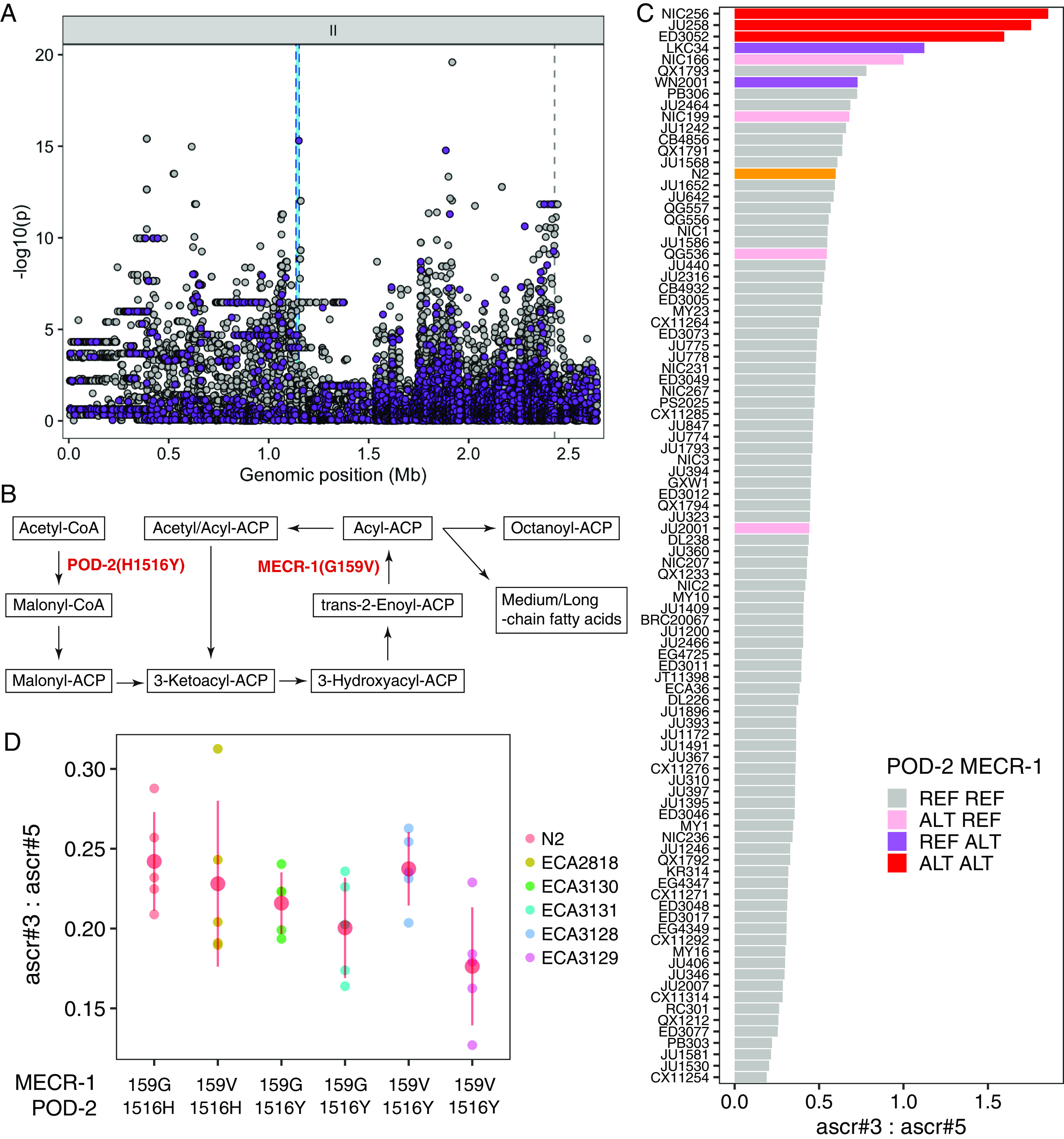
Another mtFAS gene, *pod-2,* is also associated with natural differences in ascaroside production. (*A*) Fine mapping of the ChrIIL-QTL (II:4,512-2,641,359) for the ascr#3:ascr#5 trait is shown. Each dot represents an SNV that is present in at least 5% of the 94 wild strains. The association between the SNV and ascr#3:ascr#5 trait value is shown on the *y* axis and the genomic position of the SNV is shown on the *x* axis. SNVs with high or moderate impact inferred from SnpEff are colored purple. (*B*) Schematic of the mitochondrial FA synthesis (mtFAS) pathway. Two enzymes (POD-2 and MECR-1) that harbor missense variants associated with ascaroside production variation are shown. (*C*) A bar plot for the ascr#3:ascr#5 trait value of 94 wild *C. elegans* strains. The reference N2 strain is colored orange and other wild strains are colored by the genotype of two sites (MECR-1(G159V) and POD-2(H1516Y)). (*D*) Phenotypes of POD-2 allele–replaced strains are compared with the N2 reference parental strain (159G, 1516H) and ECA2818 MECR-1–edited strain (159V, 1516H). Two independent POD-2 allele–replacement strains for each background (ECA3130 and ECA3131 for N2; ECA3128 and ECA3129 for ECA2818) were tested. On the *y* axis, values of ascr#3:ascr#5 ratio traits are shown.

To test this hypothesis, we performed a fine-mapping for the ChrIIL-QTL, which has a much smaller CI (2.6 Mb) than that of the ChrIV-QTL (6.5 Mb) that spans almost half of chromosome IV (Fig. 6*A*). In total, we analyzed the association between 28,230 genetic variants in ChrIIL-QTL with the ascr#3:ascr#5 ratio trait. The five most significantly associated variants were identified in the genes *bath-4*, *nstp-4*, *pod-2*, *T04B8.5*, and *math-7* (Dataset S3). Intriguingly, the most significantly associated variant predicted to impact gene function was in *pod-2*, an ortholog of human ACACA (acetyl-CoA carboxylase alpha). POD-2 is predicted to act upstream of MECR-1 in the mtFAS pathway (−log_10_*p* = 15.300, Fig. 6*B*). Given the low probability of randomly mapping two highly significant coding variants in two distinct genes within the same biochemical pathway from two separate QTLs, we prioritized the *pod-2* variant for further analysis. The alternative POD-2(1516Y) allele is associated with a higher ascr#3:ascr#5 ratio than that of the reference POD-2(1516H) allele (Fig. 6*C*). Furthermore, the association patterns of pairwise ratio traits are similar to those of MECR-1(G159V) variant (*SI Appendix*, Fig. S5). Most importantly, three wild strains that exhibit extremely high ascr#3:ascr#5 ratios carry alternative alleles for both genes (Fig. 6*C*).

To analyze the effects of the POD-2(H1516Y) variant and its genetic interaction with the MECR-1(G159V) variant, we generated allele-substituted strains in both the N2 and the N2 MECR-1(159V) genetic backgrounds. We found that neither the single edit of POD-2(1516H>Y) nor the double edits of both MECR-1(159G>V) and POD-2(1516H>Y) changed the ascr#3:ascr#5 ratio (Fig. 6*D*). Although these two variants are highly associated with phenotypic variation and components of the same mtFAS pathway, this result shows that neither MECR-1(G159V), POD-2(H1516Y), nor both variants are sufficient to change the ratio of ω-ascarosides to (ω-1)-ascarosides in the N2 background.

## Discussion

We explored natural variation in ascaroside pheromone production of *C. elegans* and its genetic basis. By profiling excreted metabolites across 95 wild *C. elegans* strains, we found that ascaroside pheromone bouquets differ between strains in several different ways. In the most extreme case, we found complete absence of ascaroside pheromones in the JU1400 strain likely caused by a deletion encompassing the peroxisomal β-oxidation gene *daf-22*. Similar to *daf-22(ok693)* laboratory mutants, this wild strain has lost the ability to produce short- and medium-chain ascarosides and instead accumulates long-chain precursors (Fig. 1 *E* and *F*). The natural loss of the *daf-22* gene was surprising because ascaroside pheromones are known to play key roles in the survival and reproduction of the species (4). Notably, we scanned over 500 wild *C. elegans* genomes and identified this loss only in the genome of the JU1400 strain. This strain was sampled from an urban garden in the city center of Seville, Spain, suggesting that this rare *daf-22* deletion has been maintained in a human-associated environment. Similarly, a nonsense mutation in the carboxylesterase *cest-3* in the ECA36 strain was correlated with the lack of indole ascarosides, which regulate dwelling and aggregation behaviors (8). We also found this *cest-3* variant from the genome of 28 other wild *C. elegans* strains that were sampled across Pacific regions (30), suggesting that this variant can be maintained in the natural populations.

In addition, our analysis revealed a negative correlation between the relative abundances of ω- and (ω-1)-ascarosides across many different natural strains, highlighted by their most abundant representatives, ascr#5 (ω) and ascr#3 (ω-1), which together account for more than 70% of measured ascarosides. The structural difference between ω- and (ω-1)-ascarosides, which also regulate different traits, likely arises from differences in the metabolism of their long-chain fatty acid precursors, which presumably get hydroxylated in specific positions of the chain followed by attachment of the ascarylose, producing long-chain precursors of either ω- or (ω-1)-ascarosides. Because the origin of long-chain ascaroside precursors remains unknown, we speculate that the ratio of (ω-1) to ω-ascaroside is determined by the hydroxylation of long-chain alkyl precursors (i.e., hydroxylation at either the ω or ω-1 carbon), resulting in fatty acid attachment to ascarylose at the corresponding carbon. This difference might be caused by different metabolic inputs to the ascaroside pathway or from the expression of different tailoring enzymes (e.g., cytochrome P450 oxidases), which could preferentially hydroxylate the ω or ω-1 position (34). Interestingly, we found that the five strains with the highest ascr#3:ascr#5 ratios were isolated from Europe and Africa but not from the Pacific region where *C. elegans* likely originated (35, 36). By contrast, the five strains with the lowest ascr#3:ascr#5 ratios include two strains from the Pacific region (*SI Appendix*, Fig. S7). This result suggests that the relative abundances of ω- and (ω-1)-ascarosides were reversed in some populations during the out-of-Pacific expansion of *C. elegans*, which is hypothesized to have been facilitated by human activity (37).

Our GWA mapping analysis uncovered hot spot genomic loci that underlie relative abundances of various ascarosides as well as the ascr#3:ascr#5 ratio. Two hot spot QTL on chromosome II were mapped, respectively, to loci that harbor coding variants in mtFAS pathway genes (*mecr-1* and *pod-2*) that are highly associated with the (ω-1)-to-ω-ascaroside ratio. We hypothesize that the mtFAS pathway underlies the balance between the two parallel ascaroside biosynthetic pathways, and genetic variants in the mtFAS pathway contribute to the natural differences in the usage of the two pathways. However, our allele replacement experiments in the N2 strain failed to demonstrate the causal effects of these two variants, which could be interpreted in several ways. First, the mtFAS pathway might not be involved in ascaroside biosynthesis, or at least may not be responsible for the natural variation in (ω-1)-to-ω-ascaroside ratio. Second, two variants [MECR-1(G159V) and POD-2(H1516Y)] that we edited may be neutral but linked to uncharacterized causal variants in other genes. Finally, complex genetic interactions could mask the effect of these alleles. Recently, incompatible versions of a galactose metabolic pathway were characterized in *Saccharomyces cerevisiae* (38), in which the incompatible combination of alleles of metabolic genes is not found in nature. We failed to introduce MECR-1(159G>V) edit in two wild strains but successfully generated MECR-1(159V>G) edit in the N2 background, implying that incompatible alleles of metabolic genes might be present across wild genomes of *C. elegans*. Notably, we found strong LD among three ascr#3:ascr#5 QTL (the *mecr-1* locus, the *pod-2* locus, and the ChrIV-QTL). Therefore, although even the double-edited (MECR-1(159G>V) and POD-2(1516H>Y)) strains did not display effects on the mapped trait (ascr#3:ascr#5), this result could be explained by allele(s) in unidentified genes that segregate together and exert nonadditive phenotypic effects.

Although we focused on the (ω-1)-ascaroside-to-ω-ascaroside ratio among many observed traits in this study, we also discovered natural variation in the production of individual ascaroside pheromones and their QTL. Our dataset will provide a valuable resource for future studies to characterize genes involved in pheromone production and to explore the molecular mechanisms of how genetic changes lead to the evolution of a chemical language.

## Methods

### *C. elegans* Strains and Growth.

N2 (Bristol) and wild nematode strains were maintained at 20 °C, reared on *Escherichia coli* OP50, and grown on modified nematode growth medium containing 1% agar and 0.7% agarose (NGMA) to prevent animals from burrowing (39). Wild strains were obtained from the CeNDR (30) and are available upon request. Strain information can be found in the *C*. *elegans* Natural Diversity Resource. For the analysis of staged adults, approximately 35,000 synchronized L1 larvae obtained from alkaline bleach treatment were added to 125 mL Erlenmeyer flasks containing S-Complete medium at a density of ~3,000 animals/mL. Nematodes were fed with concentrated *E. coli* OP50 and incubated at 20 °C with shaking at 180 revolutions per minute (RPM) for approximately 64 to 70 h, at which time the population was predominantly gravid adults as determined by microscopic inspection. Liquid cultures were transferred to 15 mL conical tubes and centrifuged (500 × g, 22 °C, 1 min), and the supernatant (conditioned media, *exo*-metabolome) was transferred to a fresh conical tube and snap frozen.

### Sample Preparation.

*Exo*-metabolome (conditioned media) samples were lyophilized for 24 h using a VirTis BenchTop 4 K Freeze Dryer. Dried material was directly extracted in 3 mL methanol with gentle rocking at room temperature. Following overnight extraction, the samples were centrifuged (2,750 × g, 22 °C, 5 min) in an Eppendorf 5702 Centrifuge. The supernatant was transferred to a clean 8 mL glass vial and concentrated to dryness in an SC250EXP Speedvac Concentrator coupled to an RVT5105 Refrigerated Vapor Trap (Thermo Scientific). The powder was suspended in 150 µL methanol, vortexed vigorously for 30 s, and sonicated for 5 min. The suspension was transferred to a 1.7-mL Eppendorf tube and centrifuged (18,000 × g, 22 °C, 5 min), and the clarified supernatant was transferred to HPLC vials and analyzed directly by HPLC-HRMS, see below.

### HPLC-HRMS.

Liquid chromatography was performed on a Vanquish HPLC system controlled by Chromeleon software (ThermoFisher Scientific) and coupled to an Orbitrap Q-Exactive High-Field mass spectrometer controlled by Xcalibur software (ThermoFisher Scientific). Methanolic extracts prepared as described above were separated on a Thermo Hypersil Gold C18 column (150 mm × 2.1 mm, particle size 1.9 μM, part no. 25002-152130) maintained at 40 °C with a flow rate of 0.5 mL/min. Solvent A is 0.1% formic acid (Fisher Chemical Optima LC/MS grade; A11750) in water (Fisher Chemical Optima LC/MS grade; W6-4); solvent B is 0.1% formic acid in acetonitrile (Fisher Chemical Optima LC/MS grade; A955-4). A/B gradient started at 1% B for 3 min after injection and increased linearly to 98% B at 20 min, followed by 5 min at 98% B, then back to 1% B over 0.1 min, and finally held at 1% B for the remaining 2.9 min to reequilibrate the column (28 min total method time). Mass spectrometer parameters were spray voltage, −3.0 kV/+3.5 kV; capillary temperature, 380 °C; probe heater temperature, 400 °C; sheath, auxiliary, and sweep gas, 60, 20, and 2 AU, respectively; S-Lens RF level, 50; resolution, 120,000 at *m/z* 200; and AGC target, 3E6. Each sample was analyzed in negative (ESI−) and positive (ESI+) electrospray ionization modes with *m/z* range 100 to 1,000.

### Metabolite Nomenclature.

Ascarosides were named using Small Molecule Identifiers (SMIDs), a search-compatible nomenclature for metabolites identified from *C. elegans* and other nematodes. The SMID database (www.smid-db.org) is an electronic resource maintained in collaboration with WormBase (www.wormbase.org); a complete list of SMIDs can be found at www.smid-db.org/browse (12).

### Identification of a Large Deletion at *daf-22* Locus in JU1400.

To identify structural variants in JU1400, we downloaded a genome assembly for JU1400 assembled with PacBio long reads (36) from NCBI (GCA_016989365.1) and called structural variants using MUM&Co (40) (version 3.8; default parameters) with the N2 genome (WS285) as a reference. MUM&Co identified a 29,011 bp deletion (II:12411041-12440052) in JU1400 relative to N2 that overlaps with *daf-22* and seven other protein-coding genes (*Y57A10C.1, Y57A10C.11, sre-27, sre-26, Y57A10C.8, dct-12,* and *Y57A10C.9*). We confirmed this deletion by aligning unassembled PacBio long reads for JU1400 (PRJNA692613) to the N2 reference genome using minimap2 (41) (version 2.17; using the parameters *-a -x map-pb*) and inspecting read coverage using IGV (42) (version 2.8.13).

#### Genetic relatedness.

A VCF file containing 963,027 biallelic SNVs from a previous study (36) was filtered for 94 wild *C. elegans* strains and converted to the PHYLIP format. The distance matrix and pseudo-rooted (ECA36) neighbor-joining tree were made from this PHYLIP file using dist.ml and the NJ function, respectively, using the phangorn (version 2.5.5) R package. The tree was visualized using the ggtree (version 1.16.6) R package.

### Heritability Calculations.

Narrow-sense heritability (*h*^2^) estimates were calculated using the phenotype data of 94 wild strains. The *A.mat* functions in the sommer R package (43) were used to generate an additive genotype matrix, from the genotype matrix used for the GWA mapping. This matrix was used to calculate the additive variance components using the sommer *mmer* function. Variance components were used to estimate heritability and SE through the *pin* function (*h^2^* ~ V1/V1 + V2) in the sommer package.

### PCA.

Phenotypic values for heritable (>10% narrow-sense heritability) relative abundance traits of 23 ascarosides were used as inputs to PCA. PCA was performed using the prcomp function in R. Eigenvectors and loadings were subsequently extracted from the object returned by the prcomp function.

### GWA Mapping.

A GWA mapping was performed for heritable (>10%) relative abundance traits of 23 ascarosides as well as the ascr#3 to ascr#5 (ascr#3:ascr#5) ratio trait using the NemaScan pipeline (44) available at https://github.com/AndersenLab/NemaScan. Genotype data were acquired from the latest VCF release (release 20210121) from the CeNDR. We used BCFtools (45) to filter variants below a 5% minor allele frequency and variants with missing genotypes and used PLINK v1.9 (46, 47) to prune genotypes using LD. The additive kinship matrix was generated from the 30,065 markers using the *make-grm* and *make-grm-inbred* functions from GCTA (48). Because these markers have high LD, we performed eigen decomposition of the correlation matrix of the genotype matrix to identify 499 independent tests (49). We performed GWA mapping using the *mlma-loco* and *fastGWA-lmm-exact* functions from GCTA. Significance was determined by an eigenvalue threshold set by the number of independent tests in the genotype matrix (49). Confidence intervals were defined as ±150 SNVs from the rightmost and leftmost markers that passed the significance threshold.

### CRISPR-Cas9 Allele Replacement.

Genome editing to make alleles *pod-2(ean229)*, *pod-2(ean238)*, *pod-2(ean239)*, *pod-2(ean240)*, *mecr-1(ean216)*, and *mecr-1(ean220)* was done using CRISPR-Cas9 and the coconversion marker *dpy-10* as previously described (50). Single-strand guide RNAs for *mecr-1* and *pod-2* were designed using the online analysis platform Benchling (benchling.com). All guides were ordered from Synthego (Redwood City, CA) and injected at 1 μM for the *dpy-10* guide and 6 μM for all others. Single-stranded oligodeoxynucleotides (ssODN) templates used for HDR were ordered from Integrated DNA and injected at 0.5 μM for the *dpy-10* ssODN and 5 μM for all others, and 5 μM purified Cas9 protein (QB3 Macrolab, Berkeley, CA) was used. Hermaphrodites were staged at L4 larval stage the day before injection, and the reagents were mixed and incubated for 1 h at room temperature prior to injection into each gonad. Injected animals were singled onto NGMA plates and allowed to lay until the next generation matured to the L4/young adult stage. Plates were screened for the *dpy-10* phenotypes of Dumpy and Roller and F1s were singled from plates with a high percentage of affected worms. F1s were allowed to lay eggs before single-animal lysis and PCR, and the products were sequenced using Sanger sequencing by MCLab Molecular Cloning Laboratories (South San Francisco, CA) with no more than one edited strain per independently injected progenitor retained for the study. All alleles were confirmed by sequencing singled offspring for at least two additional generations to confirm the accuracy of the edit sequence and the homozygosity of the line.

All genome-edited strains through CRISPR are listed below:

**Table:** 

Strain name	Description	Genotype
ECA3128	ECA2818 with *pod-2* H1516Y	*pod-2(ean229) mecr-1(ean216) II*
ECA3129	ECA2818 with *pod-2* H1516Y	*pod-2(ean238) mecr-1(ean216) II*
ECA3130	N2 with *pod-2* H1516Y	*pod-2(ean239) II*
ECA3131	N2 with *pod-2* H1516Y	*pod-2(ean240) II*
ECA2834	N2 with *mecr-1* G159V	*mecr-1(ean220) II*
ECA2818	N2 with *mecr-1* G159V	*mecr-1(ean216) II*

Oligonucleotides used for the generation of genome-edited strains are listed below:

**Table:** 

Application	Sequence
*mecr-1* G159V edit repair template	CTTCTCACAACATTCACAGTTTTGATGCCAAGGATCCGGCAAATTTGAATAACGTGCTTCCCGACAGCCGAATTGGCTCCGTTTTGAGCCACTGTGTCT**A**CTTTTTTCAGGTCGATAAAGTCTTTAA
*mecr-1* guide	CCUGAAAAAAGGAGACACAG
*pod-2* H1516Y edit repair template	TTCTCGTTCTTTGCTCCGATGACGGCAAGGATCTTCAGTCTTCCATTGCCCAACTCCTCATACACAACTTCTCCCTCAATCTGTGCCTTGT**A**CTCTCCATCAATGTAAATGTACTCGAATCCTTGTT
*pod-2* guide	UUACAUUGAUGGAGAGCACA

## Supplementary Material

Appendix 01 (PDF)

Dataset S01 (CSV)

Dataset S02 (CSV)

Dataset S03 (CSV)

## Data Availability

All datasets and code for generating figures and tables are available on GitHub (https://github.com/AndersenLab/Ce-ascr) (51).
