# Supplementary material for: Natural genetic variation in the pheromone production of C. elegans
Source: Proc Natl Acad Sci U S A. 2023 Jun 20;120(26):e2221150120. doi: 10.1073/pnas.2221150120 (PMC10293855; doi:10.1073/pnas.2221150120)
Supplement: Supplementary file 1 — Appendix 01 (PDF) [file pnas.2221150120.sapp.pdf]

Natural genetic variation in the pheromone production of *C. elegans*

Supplementary Information

Daehan Lee, Bennett W. Fox, Diana Fajardo Palomino, Oishika Panda, Francisco J. Tenjo, Emily J. Koury, Kathryn S. Evans, Lewis Stevens, Pedro R. Rodrigues, Aiden R. Kolodziej, Frank C. Schroeder*, Erik C. Andersen*

* Correspondence should be addressed to E.C.A. (erik.andersen@gmail.com) and F.C.S (fs31@cornell.edu).

Supplementary Fig. 1 | Structures of 44 ascarosides

Chemical structures of the 44 ascarosides included in the analysis.

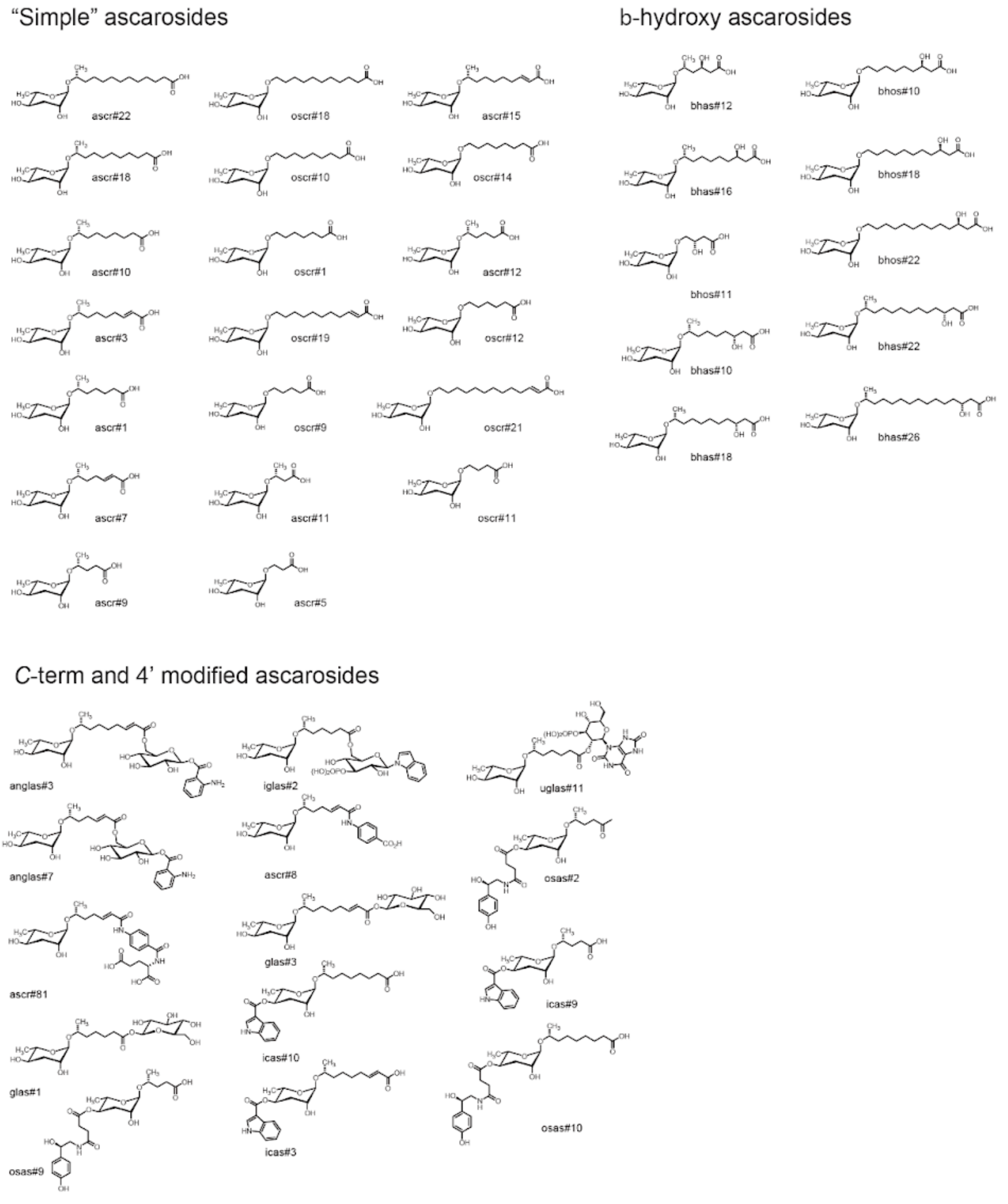

Supplementary Fig. 2 | Natural variation in the abundances of 42 ascaroside compounds
Bar plots showing relative abundances of 42 ascaroside compounds across 94 wild *C. elegans* strains, ordered by the relative abundance of each trait.

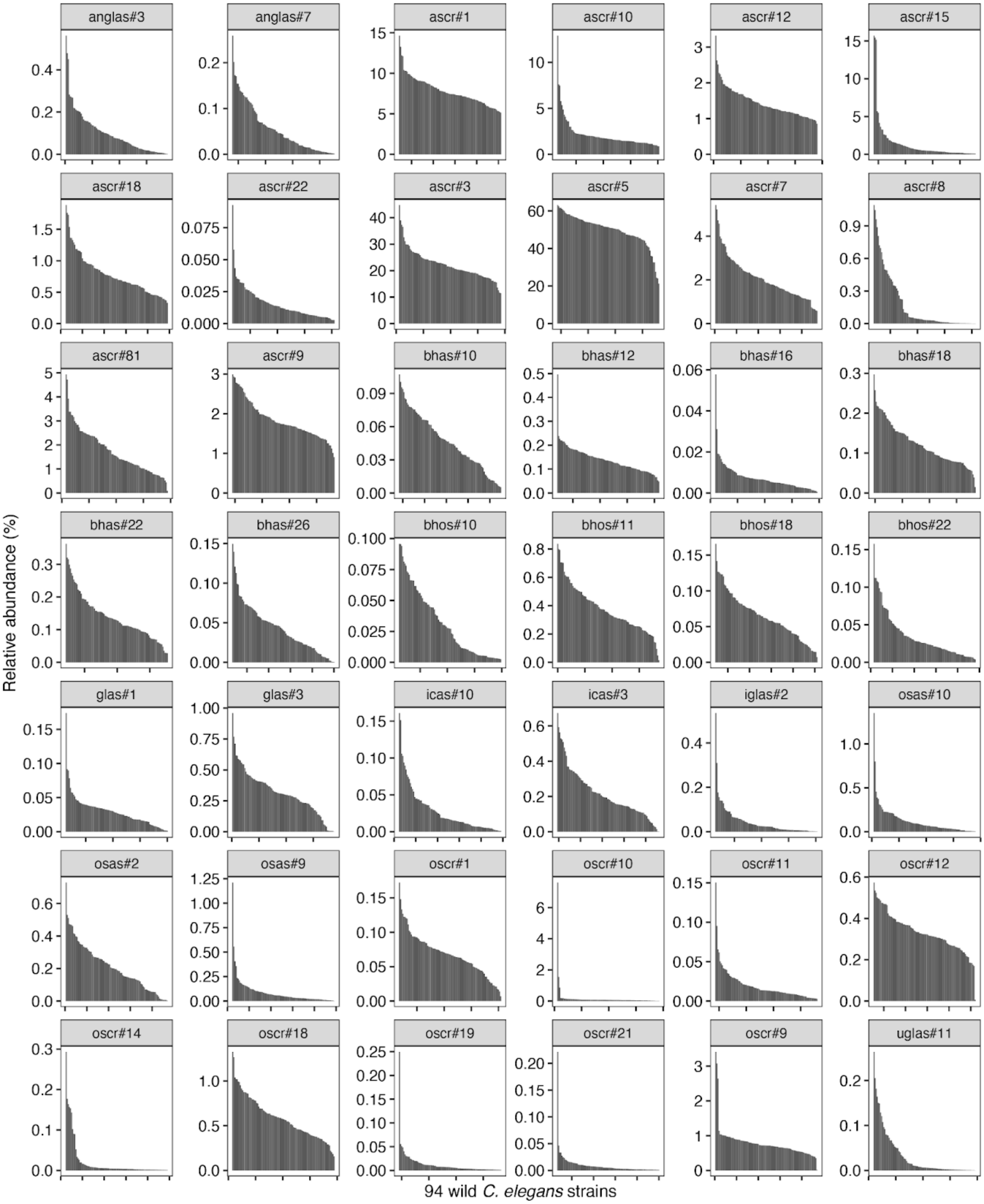

Supplementary Fig. 3 | Correlation between ascr#5 production and ascr#5 response (dauer formation)

(a) Phenotypes of the ascr#3:ascr#5 ratio (x-axis) and ascr#5-induced dauer formation frequency are shown for 61 wild *C. elegans* strains. The two traits are weakly correlated (Spearman's *rho* = 0.192). Four outlier strains for ascr#3:ascr#5 ratio traits are labeled and colored in blue. (b) Phenotypes of the ascr#3:ascr#5 ratio (x-axis) and ascr#5-induced dauer formation frequency are shown without the four outlier strains (Spearman's *rho* = 0.108).

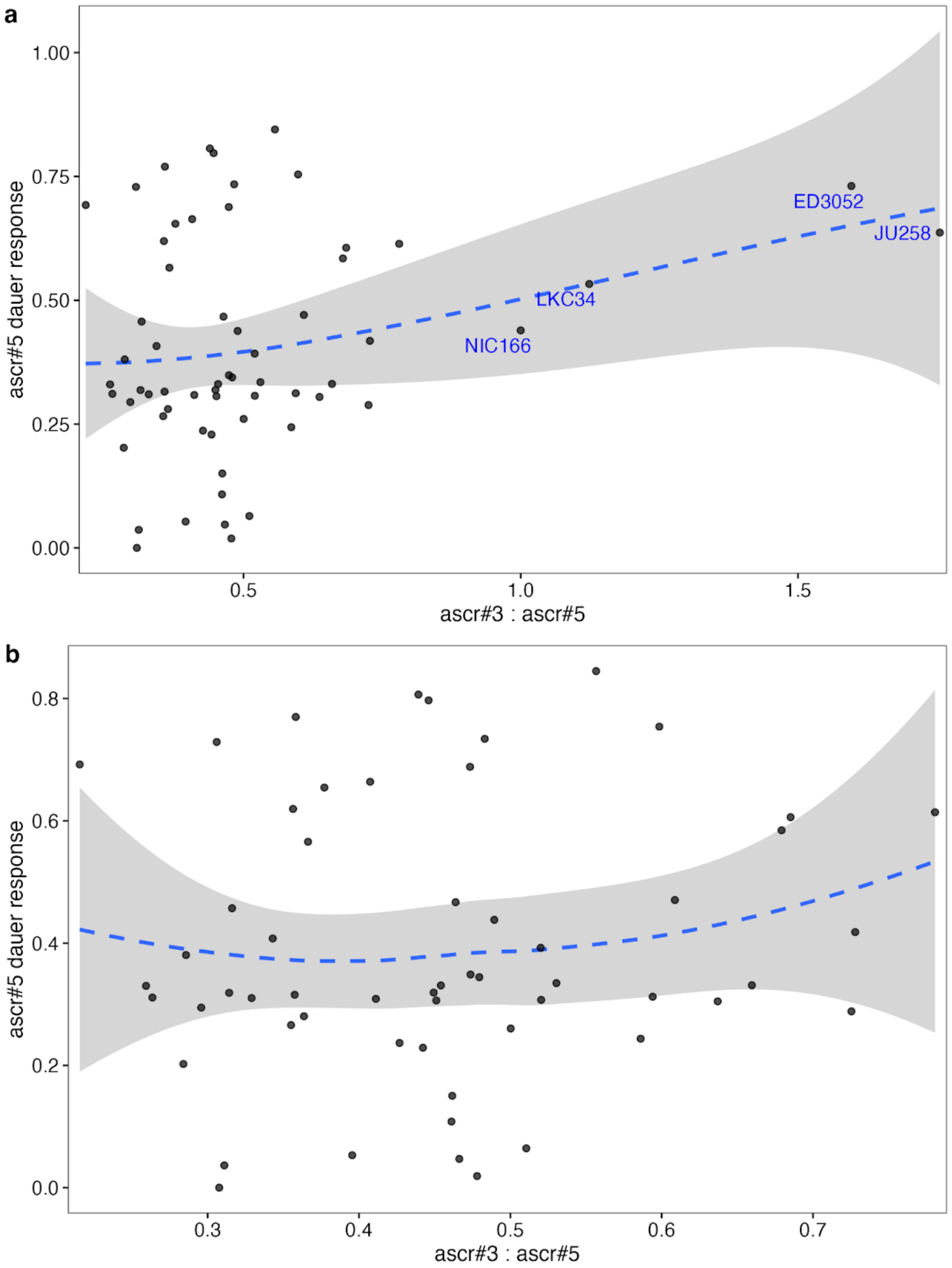

Supplementary Fig. 4 | Phenotypes of MECR-1 G159V allele-replacement strains

Phenotypes of MECR-1(G159V) allele-replacement strains are compared with the N2 reference parental strain (159G) and two wild strains with MECR-1(159V). On the y-axis, the relative ratios between ascr#3 and ascr#5 are shown.

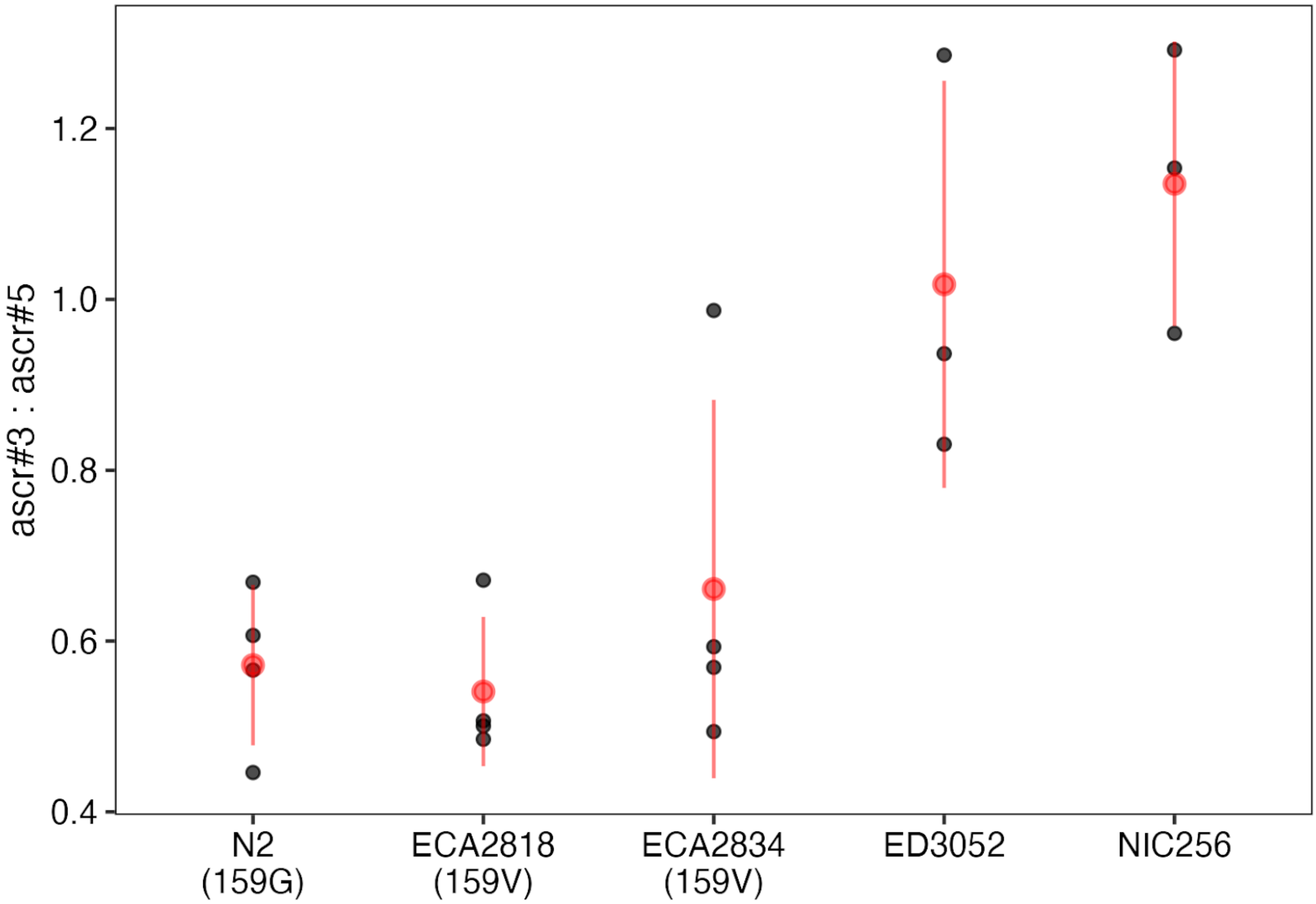

Supplementary Fig. 5 | Linkage disequilibrium among ascr#3:ascr#5 QTL
Linkage disequilibrium (r^2) values of four peak QTL markers for the ascr#3:ascr#5 trait are shown.

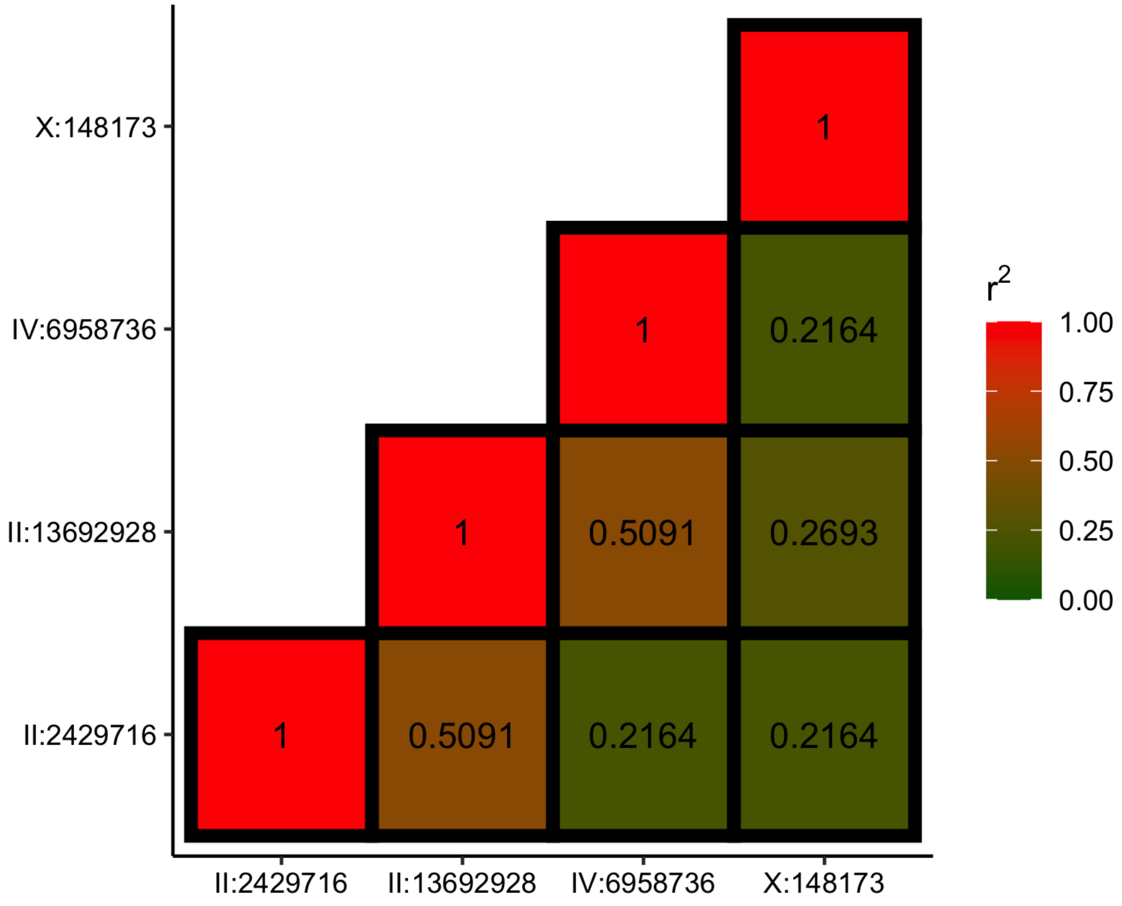

Supplementary Fig. 6 | Phenotypic variance explained by the POD-2(H1516Y) variant
A heatmap showing amounts of variance explained by the POD-2(H1516Y) variant for pairwise ratio traits of 23 ascarosides with high heritability.

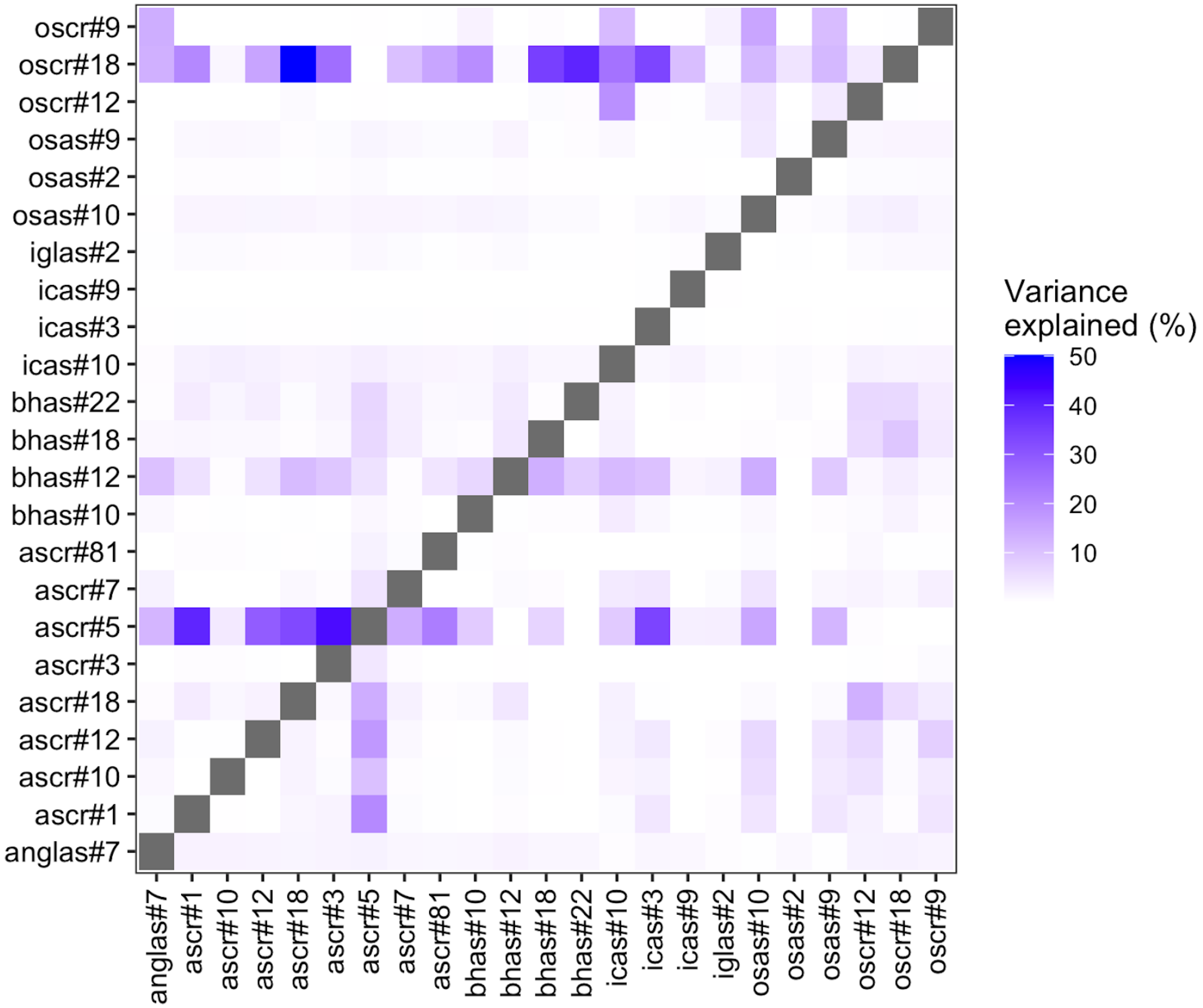

Supplementary Fig. 7 | The geographic distribution of the ascr#3:ascr#5 high and low ratio strains
Among 94 wild *C. elegans* strains, the sampling locations of 92 strains are shown here. The sampling locations of five high ascr#3:ascr#5 ratio wild strains (red, ≥1) and five low strains (blue, <0.26) are shown. The 82 wild strains with intermediate phenotypes are marked as dark gray points.

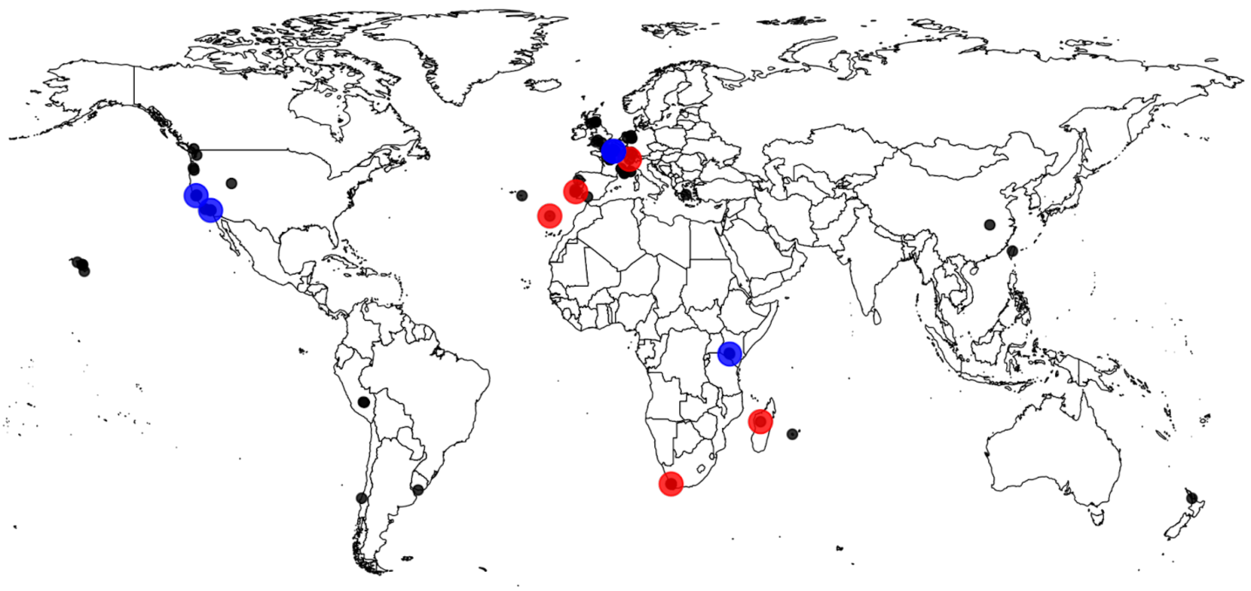

Supplementary Table 1 | Classification of 44 ascarosides

Feature	Class	N_carbons	Feature	Class	N_carbons
ascr#11	ascr(s)	4	ascr#5	oscr(s)	3
ascr#9	ascr(s)	5	oscr#11	oscr(s)	4
ascr#12	ascr(s)	6	oscr#9	oscr(s)	5
ascr#1	ascr(s)	7	oscr#12	oscr(s)	6
ascr#7	ascr(s)	7	oscr#1	oscr(s)	7
ascr#10	ascr(s)	9	oscr#14	oscr(s)	8
ascr#3	ascr(s)	9	oscr#10	oscr(s)	9
ascr#15	ascr(l)	10	oscr#18	oscr(l)	11
ascr#18	ascr(l)	11	oscr#19	oscr(l)	12
ascr#22	ascr(l)	13	oscr#21	oscr(l)	13
bhas#12	bhas	6	bhos#11	bhos	4
bhas#10	bhas	9	bhos#10	bhos	9
bhas#16	bhas	10	bhos#18	bhos	11
bhas#18	bhas	11	bhos#22	bhos	13
bhas#22	bhas	13			
bhas#26	bhas	15			
anglas#7	modified(C)	7			
ascr#8	modified(C)	7			
ascr#81	modified(C)	7			
glas#1	modified(C)	7			
iglas#2	modified(C)	7			
uglas#11	modified(C)	7			
anglas#3	modified(C)	9			
glas#3	modified(C)	9			
icas#9	modified(4')	5			
osas#9	modified(4')	5			
osas#2	modified(4')	6			
icas#10	modified(4')	9			
icas#3	modified(4')	9			
osas#10	modified(4')	9			

Supplementary Table 2 | Summary of GWA mapping

Trait	Chr	Start	Peak	End	Interval size (bp)	Variance explained	Log10p
anglas#7	I	3736	2733130	14747808	14744072	0.356921053	12.04815028
anglas#7	II	4512	1093248	6846802	6842290	0.228572941	6.749824052
anglas#7	II	13399659	14075021	14893958	1494299	0.19468924	5.619052891
anglas#7	III	373310	2125160	3787190	3413880	0.225667053	6.649273358
anglas#7	IV	4272318	13598850	17489019	13216701	0.268041954	8.189610868
anglas#7	V	593381	13756973	20654084	20060703	0.291770096	9.128340681
anglas#7	X	124570	2287865	8740424	8615854	0.336113477	11.05528198
anglas#7	X	10742782	15832204	16115328	5372546	0.212987172	6.21857424
ascr#1	II	13198273	13390616	13852723	654450	0.199305394	5.767942064
ascr#1	III	11741084	11994388	12747249	1006165	0.218405445	6.401035591
ascr#1	V	17709799	17979776	18414183	704384	0.222195319	6.53005581
ascr#1	X	1268287	2334653	7798884	6530597	0.223316347	6.568443259
ascr#10	I	92616	11607099	12296778	12204162	0.262834831	7.752245163
ascr#10	III	176496	610190	856556	680060	0.195658939	5.48507788
ascr#10	X	124570	1796524	8206669	8082099	0.247626226	7.205905297
ascr#12	II	12829939	13412550	13830067	1000128	0.187873388	5.402014285
ascr#12	X	3675721	5654087	6514350	2838629	0.187851903	5.401335315
ascr#15	X	13834552	14603523	16845496	3010944	0.210973885	5.970478244
ascr#22	IV	43186	773445	1056922	1013736	0.225775256	6.587458817
ascr#3	II	303114	2429716	2641359	2338245	0.310848244	6.01691297
ascr#3	II	12476063	13203646	13886985	1410922	0.425392871	8.688487716
ascr#5	I	695386	1233659	2260201	1564815	0.212354447	6.197417817
ascr#5	II	4512	786255	2641359	2636847	0.235902331	7.006610949
ascr#5	II	12368778	13692928	15278446	2909668	0.433081098	16.28871339
ascr#5	III	11479501	11775202	12781165	1301664	0.253949117	7.659026725
ascr#5	IV	751766	13489747	15131605	14379839	0.232898852	6.900830594
ascr#5	X	124570	2986710	6700584	6576014	0.243249564	7.268690917
ascr#5	X	10603327	12636855	13504734	2901407	0.195723586	5.652279783
ascr#7	II	2739775	2867991	3096129	356354	0.212968229	6.217940367
ascr#7	II	12368778	12716976	13298659	929881	0.203992488	5.920724667
ascr#81	II	4013239	4492510	6846802	2833563	0.188183858	5.411829173

ascr#81	II	13298548	15081782	15278446	1979898	0.203178	5.894057439
ascr#81	III	11727315	12525622	13323719	1596404	0.240632753	7.17480245
ascr#81	IV	3981501	13598850	15830951	11849450	0.376011609	13.01582251
ascr#81	V	3852565	12897665	20909920	17057355	0.230052293	6.801287943
ascr#81	X	124570	2287865	8468772	8344202	0.278411575	8.592593798
ascr#81	X	10402729	12898474	14134472	3731743	0.205360013	5.965612216
ascr#9	X	124570	867999	8196736	8072166	0.201037248	5.824204162
bhas#12	III	11479501	11801670	13775378	2295877	0.197417124	5.706851883
bhas#12	V	639357	1648854	2121953	1482596	0.193271526	5.573636782
bhas#18	II	4512	1304965	2641359	2636847	0.20995443	6.117453264
bhas#18	II	12542769	13692928	13886985	1344216	0.268616618	8.21165837
bhas#18	IV	1784821	3579831	13994964	12210143	0.197750073	5.717604495
bhas#18	X	10644948	11384708	13555358	2910410	0.208321824	6.063311369
bhas#22	II	4512	1304965	1819726	1815214	0.196040546	5.662478844
bhas#22	II	12542769	13692928	13852723	1309954	0.219786155	6.447905139
bhas#22	IV	1670573	2053138	12976479	11305906	0.244592202	7.317100755
bhos#22	IV	1489441	1658768	4903773	3414332	0.206928314	6.017260795
bhos#22	V	16774869	16912293	17723744	948875	0.190747949	5.493148752
icas#10	X	12993889	13280531	14746716	1752827	0.324310354	7.034667983
icas#3	IV	1458400	2153686	17187855	15729455	0.259176934	7.853617666
icas#3	X	4114709	5020050	5834064	1719355	0.191589274	5.519931225
icas#3	X	9782566	11067399	12958823	3176257	0.243679952	7.284191297
icas#9	II	4512	287101	930896	926384	0.23459298	6.822812808
icas#9	II	3376344	3805135	5699108	2322764	0.205637608	5.857879705
icas#9	IV	1020003	1196322	3699373	2679370	0.265917324	7.946541235
icas#9	X	4099789	11067399	12388111	8288322	0.278051744	8.406627526
osas#10	IV	1489441	15194433	16785392	15295951	0.220790754	6.354586574
osas#10	X	1499830	8910902	15347272	13847442	0.256054219	7.583086688
osas#2	I	94720	3951912	13659314	13564594	0.259253902	7.856501184
osas#2	II	11998593	13709164	14893958	2895365	0.227210457	6.702591881
osas#2	III	10477290	11650544	13464737	2987447	0.240371153	7.1654496
osas#2	IV	13180663	15550884	16120945	2940282	0.217812207	6.380944254
osas#2	V	1583467	13965981	19804412	18220945	0.213860232	6.247817825
osas#2	X	388657	2362150	8740424	8351767	0.244905162	7.328407881
osas#2	X	12921258	14341555	16241564	3320306	0.195231967	5.636478272

osas#9	IV	9103306	15559488	16820852	7717546	0.214502498	6.146323686
osas#9	X	124570	4960069	17379408	17254838	0.227130596	6.567728384
oscr#12	II	12985307	13204800	13597625	612318	0.229014473	6.765166475
oscr#12	III	11507368	11801670	12028729	521361	0.193085092	5.567675418
oscr#14	X	13179717	13882309	14622118	1442401	0.228300473	6.074616735
oscr#18	I	1738955	12959042	15064788	13325833	0.261215584	7.930206146
oscr#19	X	16309679	16879447	17536376	1226697	0.191635451	5.467732405
oscr#21	II	12910581	13692928	13852955	942374	0.199129391	5.706045529
oscr#9	I	7830004	12302651	13784685	5954681	0.229639502	6.585974782
oscr#9	III	11816876	12374645	13775378	1958502	0.20371408	5.73826383
uglas#11	II	13175817	13358149	13705977	530160	0.200120674	5.737871785
uglas#11	X	14477147	15104579	15590617	1113470	0.200843107	5.76111123
ascr#3: ascr#5	II	4512	2429716	2641359	2636847	0.360079666	6.831116714
ascr#3: ascr#5	II	12422412	13692928	13915254	1492842	0.717846993	14.2504631
ascr#3: ascr#5	IV	4212020	6958736	10752480	6540460	0.450031818	6.655576285
ascr#3: ascr#5	X	124570	148173	4987366	4862796	0.356242553	6.169168913
